# Wound fluids collected postoperatively from patients with breast cancer induce epithelial to mesenchymal transition but intraoperative radiotherapy impairs this effect by activating the radiation-induced bystander effect

**DOI:** 10.1038/s41598-019-44412-y

**Published:** 2019-05-27

**Authors:** Katarzyna Kulcenty, Igor Piotrowski, Karolina Zaleska, Mateusz Wichtowski, Joanna Wróblewska, Dawid Murawa, Wiktoria Maria Suchorska

**Affiliations:** 1 Radiobiology Laboratory, Greater Poland Cancer Centre, 61-866 Poznań, Poland; 2 Department of Electroradiology, Poznań University of Medical Sciences, 61-701 Poznań, Poland; 3 Department of Breast Cancer Surgery, Greater Poland Cancer Centre, 61-866 Poznań, Poland; 4 Department of Pathology, Poznan University Medical Sciences and Greater Poland Cancer Center, Poznan, Poland; 5 Department of Surgery and Oncology, University of Zielona Góra, Zielona Góra, Poland; 6 General and Minimally Invasive Surgery, Poland Baptism Monument Hospital, Gniezno, Poland

**Keywords:** Breast cancer, Cancer microenvironment

## Abstract

Wound fluids (WF) are believed to play a role in the local recurrences by inducing an inflammatory process in scar tissue area. Given that most local relapse in primary breast cancer patients occur within the scar tissue area, researchers have investigated whether localized radiotherapy, such as intraoperative radiotherapy (IORT), could be more effective than postoperative RT in inhibiting local tumor recurrence. The epithelial-mesenchymal transition (EMT) program plays a critical role in promoting metastasis in epithelium-derived carcinoma. Given this background the main aim of the present study was to determine the mechanisms by which IORT decreases the tumorigenic potential of WF. We assumed that postoperative fluids from patients would activate the radiation-induced bystander effect (RIBE) in treated cells, thus altering the tumor microenvironment. To confirm this hypothesis, WF collected from patients after breast conserving surgery (BCS) alone, after BCS followed by IORT treatment or WF from BCS patients together with RIBE medium were incubated with MCF7 and MDA-MB-468 cells. Changes in the CSC phenotype, in EMT program and potential to migrate were performed to determine the possible role of WF on the migration of breast cancer cells. Our findings show that wound fluids stimulate the CSC phenotype and EMT program in breast cancer cell lines. This effect was partially abrogated when the cells were incubated in wound fluids collected from patients after breast-conserving surgery followed by IORT. Additionally, we confirmed the role of radiation-induced bystander effect in altering the properties of the WF to induce the CSC phenotype and EMT program.

## Introduction

Breast-conserving surgery (BCS) followed by fractionated external beam radiation therapy (EBRT) is a standard treatment approach for early breast cancer. Intraoperative radiotherapy (IORT), which is administered in a single dose during surgery, is an emerging alternative^1^. The rationale behind IORT is that a single high dose of radiation can potentially prevent residual tumor cell repair and proliferation that may occur in fractionated radiotherapy delivery; IORT should improve the biological effect of radiotherapy^2,3^.

The precise effects of surgery and radiation on the tumor bed are still largely unknown. However, many researchers have pointed out that the inflammation and wound healing process that occurs post-surgically could stimulate residual cancer cell growth^4–6^. The presence of inflammatory factors following surgical excision of the primary tumor has been confirmed in mouse serum^7^ and, more recently, in drainage fluids (wound fluids [WF]) harvested post-operatively in patients treated for breast cancer^8,9^. Besides the direct effects of radiation on cancer cells, radiotherapy can also modify the tumor microenvironment, which could thereby affect tumor development^10,11^. The non-targeted effects of radiation are mediated by gap junctions communications and immune-signaling mediated mechanisms, which affect surrounding non-irradiated cells^12^. This non-targeted effect in unirradiated cells is known as the radiation-induced bystander effect (RIBE), which is mediated by gaps junctions between the cells and through mediators released from irradiated cells, mainly cytokines and chemokines^13,14^. Belletti *et al*. analyzed how normal and breast carcinoma cells are affected by WF obtained from patients after BCS plus IORT (the TARGIT clinical trial)^8^. Using proteomic analyses, the authors showed that IORT significantly modified the protein expression profile of the WF^8^, with downregulation of various cytokines involved in tumor cell growth and motility in the IORT group. These experiments proved that TARGIT could have an antitumor effect, probably through the growth factors and cytokines present in WF. We have previously reported, that WF collected 7 days after surgery from patients who underwent surgery alone, and those who received IORT after surgery affect the stem cell phenotype in breast cancer cell lines. Moreover we revealed a lower stimulation of CSC phenotype in IORT treated patients^15^.

Inflammation has also been proven to be a potent inducer of epithelial to mesenchymal transition (EMT) in tumor cells^16^. Neoplastic epithelial cells induced to undergo EMT initiate a cascade of tumor cell invasion and enter the vasculature and go on to form metastatic lesions. The EMT process is reversible, and cells can revert from mesenchymal to epithelial cells in a process known as mesenchymal to epithelial transition (MET) after metastatic colonization^17,18^. Recent findings also indicate that the EMT process confers mesenchymal cells stem cell features that enable carcinoma cells to metastasize at secondary sites^19^.

Given this background, the aim of the present study was to examine the effect of WF obtained from patients after BCS alone (WF group) and after BCS plus IORT (RT-WF group) 48 hours after surgery on cancer stem cell (CSC) phenotype and induction of the EMT program in two histopathologically different breast cancer cell lines (luminal A MCF7 and basal MDA-MB-468). Our previously published data indicated the changes of biological activity between surgical fluids from BCS patients and BCS patients followed by IORT treatment and the composition of those fluids. Based on these data we hypothesized that this effect might be in part caused by RIBE factors secreted by cells irradiated by IORT to tumor bed^20^. To better characterize the results obtained after IORT, we analyzed the impact of the RIBE medium on breast cancer cells incubated with WF (WF + RIBE group). We believe that the present findings will contribute both to a better understanding of the role of wound fluids in tumor progression and to the impact of non-targeted radiation effects on epithelial to mesenchymal process.

## Materials and Methods

### Surgical WF collection

WF were collected from breast cancer patients 48 hours after BCS at the Greater Poland Cancer Centre in Poznan, Poland. Following tumor resection, one group of patients (denominated the RT-WF group) underwent IORT boost (up to 10 Gy) to the tumor bed (Mobetron, IntraOPMedical Inc. Santa Clara, USA). The second group of patients did not receive IORT (WF group). WF were collected from drainage bottles, centrifuged for 25 min at 300 x g at 4 °C, sterile filtered and stored at −80 °C. The study was approved by the Bioethics Committee of Poznań University of Medical Sciences, study number 756/16.

RT-WF group consisted of 22 patients with an age at diagnosis 58,3 ± 11,3, and WF group of 21 patients with an age 59,6 ± 10,6. Patients characteristics are shown in Supplementary Table 1.

### Cell culture

Experiments were performed on two breast cancer cell lines with different histopathological profiles: MCF7 luminal A subtype (Er/PgR+; HER2/Neu-) and MDA-MB-468 basal subtype (Er/PgR-; human epidermal growth factor receptor 2 (HER2)/Neu-). Both cell lines were obtained from the American Type Culture Collection (ATCC, Rockville, MD) and cultured according to ATCC instructions in a humidified atmosphere with 5% carbon dioxide at 37 °C (BINDER, Germany). Cell were incubated with 10% of WF, RT-WF, RIBE or WF + RIBE (10% WF + 10% RIBE) in Dulbecco’s Modified Eagle’s Medium (DMEM, BIOWEST, UT) as described previously^15,20^ at varying time courses depending on the experiment. Control cells were cultured in standard medium (10% FBS in DMEM) under the same conditions.

### Radiation induced bystander effect medium

MCF7 and MDA-MB-468 cell lines were grown in standard culture conditions for 24 h. The culture media was replaced with a fresh medium, and the cells were exposed to 10 Gy irradiation. The media were collected 24 h after irradiation. The harvested medium was filtered through a 0.22 µm MILLEX GP filter (Merck,) and stored at −80 °C for further use.

### RNA isolation and RT-PCR

Total RNA was isolated 48 hours after stimulation with the study fluids using Direct-zol™ RNA MiniPrep (Zymo Research, CA) according to the manufacturer’s instructions. RNA was eluted in 25 µl of DEPC-treated H_2_O (Sigma, Aldrich, Merck KGaA, Darmstadt, Germany), analyzed in 1.5% agarose gel for RNA integrity, and stored at −80 °C until further analyses. One µg of total RNA was then subjected to reverse transcription using the iSCRIPT kit (BioRad, CA) according to manufacturer’s instructions.

### Real-time quantitative PCR (RT-qPCR)

The expression of markers associated with EMT was analyzed using specific primers and UPL probes designed using RealTime Ready Assay Design and Roche Probes Master kit (Roche, Basel, Switzerland) on LightCycler96 (Roche, Basel, Switzerland) according to the manufacturer’s instructions. The qPCR reaction was performed on WF from 20 patients for each group and each experiment was performed twice.

### Western blot and antibodies

Western blot analysis was performed using 12 pooled WF (from 12 different patients) for each group. Protein lysates isolated 4 days after stimulation of breast cancer cell lines with the study fluids were prepared in RIPA Buffer (Sigma Aldrich; Merck KGaA, Darmstadt, Germany) containing a protease cocktail inhibitor (Sigma Aldrich; Merck KGaA, Darmstadt, Germany). Protein concentrations were measured using BCA assay (Thermo Scientific Pierce, Rockford, IL) and 50 µg of protein was run using MINI PPROTEAN TGX 4–15% gradient gel (BioRad, CA). Proteins were transferred to a PVDF membrane (BioRad, CA) using the Trans-Blot TURBO transfer System (BioRad, CA) and then the membrane was blocked with 5% dry fat milk in 1 × TBST. Primary and secondary antibody incubation was performed according to manufacturer instructions (Cell Signaling Technology, MA). Blots were labeled with Western Bright Quantum (Advansa, Chaam, Netherlands) and imaged using the ChemiDoc Touch Imaging System (BioRad, CA). Primary antibodies were used in concentrations: E-cadherin 1:1000, N-cadherin 1:1000, SNAI1 1:1000 – all from EMT Antibody Sampler Kit (#9782, Cell Signaling Technology, MA); β-actin 1:100 (sc-130656, Santa Cruz, TX).

### Flow cytometry

MCF7 and MDA-MB-468 cell lines were incubated with the study fluids (20 different WF for each group) for four days and then detached using Accutase (BioWest, France). Cells were washed with 5% BSA in 1x PBS and labeled with fluorochrome conjugated antibodies: Alexa Fluor® 647 CD324 (E-Cadherin, CDH1) (BD 563571), Alexa Fluor® 488 CD325 (N-Cadherin, CDH2) (BD 562119), APC CD44 (BD 559942), PE CD24 (BD 555428) (Becton Dickinson, CA) by incubating at 4 °C for 30 minutes in the dark. Cells were washed twice with 5% BSA in 1x PBS, fixed with BD Cell FIX (Becton Dickinson, CA) and analyzed on a BD Accuri C6 Flow Cytometer. Unstained, isotype, and single antibody controls were performed for each cell line. All data were analyzed using FlowJo v10 analysis software.

### ALDEFLUOR assay

ALDH activity was analyzed as previously described^15^. Shortly, cells were treated for 4 days with WF, RT-WF and WF + RIBE fluids, detached with Accutase (BioWest, France) and subjected to ALDEFLUOR Assay based on manufacturer protocol. For each sample negative controls were incubated under the same conditions with diethylaminobenzaldehyde (DEAB).

### Scratch assay

*In vitro* scratch assays were performed on the MCF7 and MDA-MB-468 cell lines as previously described^21^. Briefly, cells were incubated for 48 hours with the study fluids (12 different WF for each group in duplicate). Then, a cell monolayer was scraped using a 200 µl pipet tip, washed twice with Phosphate Buffer Saline (PBS, BIOWEST, UT), and a culture medium with the study fluids was added. Images were taken every 2 h using an inverted light microscope. The scratch assay was analyzed using ImageJ software and the v.6 GraphPad Prism program (GraphPad Software, Inc., La Jolla, CA, USA).

### Statistical analysis

Statistical analyses were performed using the GraphPad Prism software program, v.6 (GraphPad Software, Inc., La Jolla, CA, USA). Data were examined using one-way anova analysis with Tukey’s post-hoc test. P < 0.05 was considered to indicate a statistically significant difference.

### Ethical statement

Sample collection was approved by the Bioethics Committee of Poznań University of Medical Sciences, study number 756/16. The research was performed in accordance with the Bioethics Committee guidelines. The informed consent was obtained from patients.

## Results

### Cancer stem cell phenotype after WF stimulation

The main aim of this study was to analyze the role of postoperative WF collected from patients after IORT on the CSC population and on the invasiveness of breast cancer cells in the context of the indirect radiation response (bystander effect). To examine this effect, we first analyzed the changes in the CSC phenotype of breast cancer cell lines after incubation with 3 different groups of WF: (1) RT-WF, defined as WF collected from patients after BCS plus IORT treatment, (2) WF, defined as WF collected from patients after BCS alone, and (3) WF + RIBE, defined as WF collected from patients after surgery alone with the addition of the RIBE medium. In order to analyze the effect of RIBE without any perturbing factor, we decided to perform the analysis on the medium collected from the coresponding cells. In a previous study^15^, we proved that surgical WF from the RT-WF and WF groups obtained 7 days after the surgery (late WF) affect the putative stem cell phenotype as determined by CD44^+^/CD24^−/low^ and high aldehyde dehydrogenase 1 (ALDH1) activity. In that study, we observed a lower stimulation of the stem cell phenotype after IORT compared to fluids harvested after surgery alone^15^.

**Figure 1:**
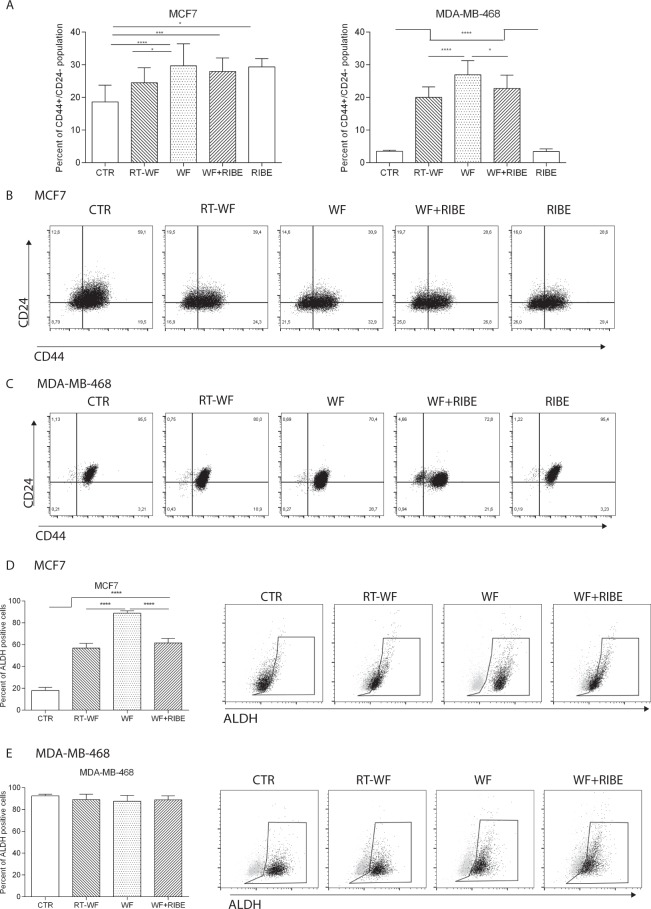
CSC phenotype after wound fluid stimulation. MCF7 and MDA-MB-468 cell lines were incubated with WF, RT-WF, WF + RIBE or RIBE fluids for 4 days and subjected to flow cytometry analysis for CD44 and CD24 antigens. (**A**) The graphs represents a shift between CD44 and CD24 antigens expressed on the surface of MCF7 and MDA-MB-468 cell lines after incubation with WF. Graphs shows means ± standard deviation of CD44/CD24 phenotype with statistical significance only for the cancer stem cell phenotype group (CD44^+^/CD24^low/−^) *p < 0.05; **p < 0.01; ***p < 0.001, ****p < 0.0001. (**B**,**C**) Dot plots represents representative results of cancer stem phenotype (CD44^+^/CD24^low/−^) changes in MCF7 (**B**) and MDA-MB-468 (**C**) cell lines after WF stimulation, (**D**,**E**) Graphs and dot plots (grey - control DEAB, black – sample) represents the percent of ALDH activity in MCF7 (**D**) and MDA-MB-468 (**E**) cell line after incubation with WF.

Given these findings, in the present study we wanted to evaluate whether WF collected 48 hours after surgery (early WF) would trigger a similar effect. For the experiments, two breast cancer (BC) cell lines with different histopathological characteristics were chosen: luminal A subtype of BC–MCF7, and basal subtype of BC–MDA-MB-468. After incubation with postoperative fluids, the CD44^+^/CD24^−^ populations were analyzed (Fig. 1A–C). Induction of the CD44^+^/CD24^−^ phenotype was observed in both cell lines after stimulation with RT-WF, WF, and WF + RIBE, but particularly in the basal MDA-MB-468 cell line (Fig. 1A right, C). Furthermore, we observed significant differences between the various WF groups in the stimulatory effect on the CSC phenotype: in the MCF7 cell line, the contribution of the CD44^+^/CD24^−^ population was significantly higher in WF-treated cells (29.7% ± 6.7) compared to control cells (CTR) without WF stimulation (18.8% ± 5.2) and RT-WF (24.5% ± 4.6) treated cells. However, no statistically significant changes were observed in WF + RIBE treated cells (27.95% ± 4.1) (Fig. 1A,B). In the MDA-MB-468 cell line, the greatest induction of the CSC phenotype was observed in WF-treated cells (27.0% ± 4.4). In the RT-WF and WF + RIBE groups, the induction rate was similar (20.0% ± 3.2 and 22.7% ± 4.1, respectively) and much higher when compared to control cells (3.5% ± 0.4). In the MDA-MB-468 cell line, in contrast to the MCF7 cell line, the differences between the CSC phenotype in RT-WF- and WF + RIBE-stimulated cells were not statistically significant.

Moreover to validate those data, ALDH1 activity of MCF7 and MDA-MB-468 cells after incubation with wound fluids was analyzed. The ALDH1 activity was evaluated in human breast cancer cell lines using the ALDEFLUOR assay. We confirmed the higher ALDH activity in MCF7 cell lines after stimulation with all 3 groups of fluids compared to CTR cells. The ALDH activity was significantly higher in WF group compared to RT-WF and WF + RIBE (Fig. 1D). We did not see any changes in ALDH activity in MDA-MB-468 cell line, probably due to very high ALDH activity in CTR cells (95% of ALDH positive cells).

These results show that postoperative WF stimulates a CSC phenotype in MCF7 and MDA-MB-468 cell lines, and that this effect in partially abrogated in both cell lines in WF collected from patients who underwent IORT (RT-WF) and in cases in which WF-treated cells were also incubated in the RIBE medium (but only in the MDA-MB-468 cell line).

### Wound fluids collected from patients after BCS affects the EMT program in MCF7 and MDA-MB-468 cell lines

**Figure 2:**
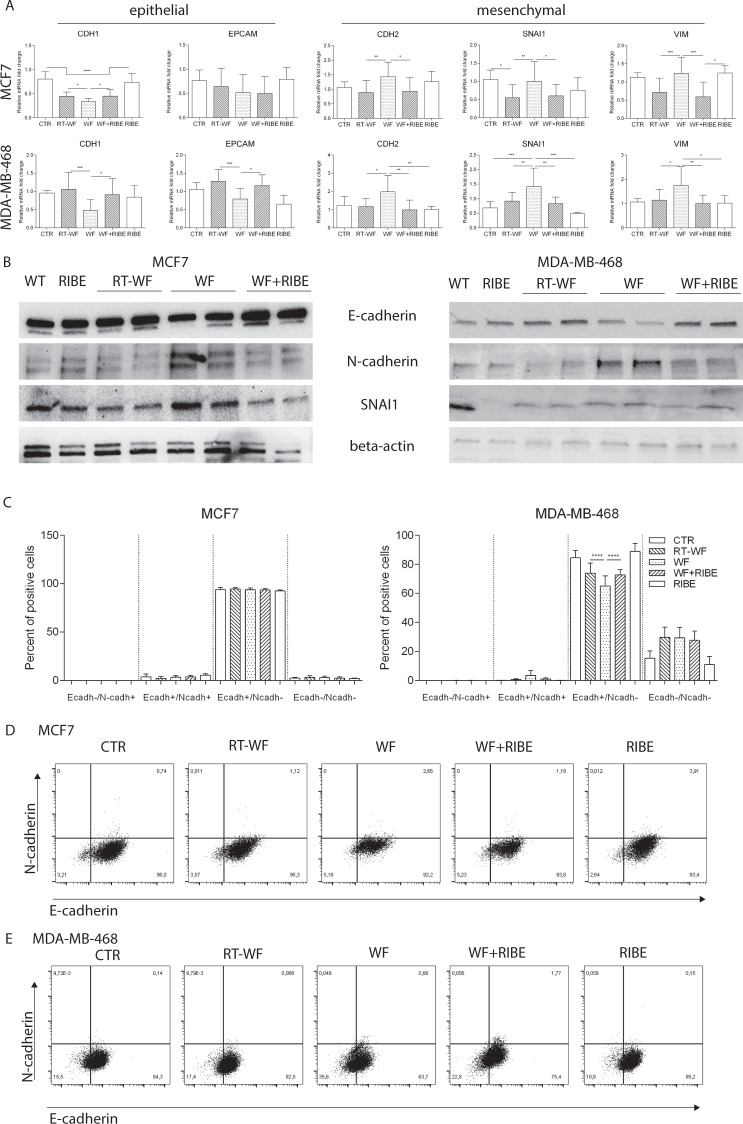
Wound fluids collected from patients after breast conserving surgery affect the EMT program in MCF7 and MDA-MB-468 cell lines. MCF7 and MDA-MB-468 cell lines were incubated with WF, RT-WF, WF + RIBE or RIBE fluids for 2 days (transcript analysis) or 4 days (protein analysis) based on timeline experiments (data not shown). The epithelial to mesenchymal transition was estimated both at the transcript and the protein level. (**A**) The level of epithelial and mesenchymal markers was determined by RT-qPCR in both cell lines incubated in the four fluids. Graphs represents relative mRNA fold changes ± standard deviation. (**B**) Changes in selected epithelial and mesenchymal markers in both cell lines incubated with the analyzed fluids were also confirmed at the protein level. Results of Western Blot are presented in duplicate for 12 pooled wound fluids for each group. (**C**) Graphs represents the shift between CDH1 and CDH2 expression (flow cytometry) in both cell lines incubated with the study fluids. *p < 0.05; **p < 0.01; ***p < 0.001, ****p < 0.0001. (**D**,**E**) Dot plots represents representative results of CDH1+/CDH2− population changes in MCF7 (**D**) and MDA-MB-468 (**E**) cell lines after wound fluids stimulation.

Induction of EMT in tumor cells may be due to the changes of a tumor microenvironment (i.e., inflammatory cells infiltrating the tumor) and CSCs present in that tumor cell microenvironment^22^. Given this situation, we wondered whether WF could induce the EMT program and whether IORT and RIBE play an inhibitory role in this process. To examine this question, we incubated MCF7 and MDA-MB-468 cell lines in the aforementioned fluids and assessed EMT markers both at the transcript (Fig. 2A, Supplementary Table 2) and protein levels (Fig. 2B–E). In the MCF7 cell line, incubation in the three fluid groups decreased the expression of epithelial marker CDH1 compared to control cells and RIBE treated cells (Fig. 2A, Supplementary Table 2). Notably, CDH1 expression was statistically lower in the WF group versus the other two groups, and there were no differences between the RT-WF and WF + RIBE groups. No statistical differences between the three groups were observed in the other epithelial marker (EPCAM) (Fig. 2A, Supplementary Table 2). In the MDA-MB-468 cells line, epithelial marker (CDH1 and EPCAM) expression was similar after incubation with WF: that is, expression of these markers was not altered by incubation in RT-WF or WF + RIBE compared to control cells. By contrast, expression of both of these markers decreased significantly after incubation in WF. Given this reduction in epithelial marker expression post-incubation, we speculated that those cells (i.e., those incubated in WF) may present an elevated level of mesenchymal markers. For this reason, we analyzed the expression of 3 mesenchymal markers (CDH2, SNAI1, and VIM) after incubating the MCF7 and MDA-MB-468 cell lines in the three different fluids. In both cell lines we observed a statistically elevated level of CDH2, SNAI1 and VIM after WF stimulation compared to both the RT-WF group and the WF + RIBE group (Fig. 2A, Supplementary Table 2). The levels of these mesenchymal markers either remained unchanged or without a significant reduction after RT-WF and WF + RIBE incubation compared to control cells and RIBE treated cells (Fig. 2A, Supplementary Table 2). Gene expression analysis confirmed that postoperative WF collected from patients after surgery alone undergo EMT and that this effect is more pronounced in the MDA-MB-468 cell line.

To confirm the presence of the EMT program at the protein level in both cell lines after WF stimulation, we performed western blot analysis (Fig. 2B) followed by flow cytometry analysis to assess changes in CDH1 and CDH2-positive cells (Fig. 2C–E). In the MCF7 cell line no clear differences in CDH1 and CDH2 protein levels were observed between control cells, RIBE, RT-WF, WF, and WF + RIBE groups on the western blot analysis (Fig. 2B) nor on the flow cytometry for CDH1^+^/CDH2^−^ cell populations (Fig. 2C,D): (control cells = 93.7% ± 2.3. RT-WF = 94.4% ± 1.7, WF = 93.7% ± 1.7, WF + RIBE = 93.6% ± 1.3, RIBE = 92.4% ± 1.0). By contrast, MDA-MB-468 cell lines shows a broad spectrum of protein level changes after incubation with the WFs. For CDH1, a decrease in protein levels was observed in WF-treated cells versus both control and RT-WF and WF + RIBE treated cells. The lowest level of CDH2 mesenchymal marker expression was observed in the RT-WF group. Emergence of the SNAI1 marker was observed in RT-WF, WF, and WF + RIBE treated cells, with the highest expression in the WF group (Fig. 2B). Flow cytometry confirmed the changes in EMT after WF stimulation (Fig. 2C,E). WF-treated cells presented the greatest changes from epithelial to mesenchymal phenotypes. The CDH1^+^/CDH2^−^ population level was lowest in WF-treated cells (65.1 ± 6.9) compared to the other variants. The CDH1^+^/CDH2^−^ population level was similar in the RT-WF and WF + RIBE treated cells and significantly higher than in the WF group.

These findings provide valuable data on induction of EMT markers by WF and in support for an inhibitory role for IORT and RIBE on these changes.

### Reduction of the tumorigenic potential of fluids collected from patients after breast-conserving surgery plus IORT measured by their potential to migrate in in vitro culture

**Figure 3:**
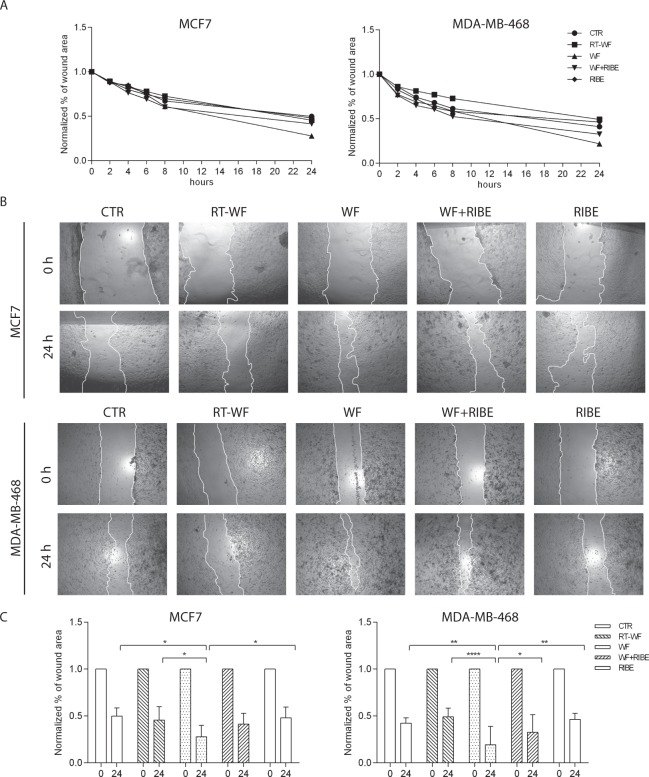
Reduction of tumorigenic potential of post-operative fluids collected from patients after breast cancer conserving surgery followed by intraoperative radiation therapy, as measured by their potential to migrate in the *in vitro* MCF7 and MDA-MB-468 cell line cultures. To determine the effect of wound fluids on tumorigenic potential (cell migration capacity), scratch assays were performed on two breast cancer cell lines: MCF7 and MDA-MB-468. (**A**) Graphs show the normalized (to t = 0 h) percent of wound healing at different time points after initiation of a scratch: 0 h, 2 h, 4 h, 6 h, 8 h and 24 h. The data represent means ± SD from 12 wound fluids for each group. (**B**) Representative pictures showing the scratch at t = 0 h and t = 24 h with (RT-WF, WF, WF + RIBE, RIBE) and without (CTR) wound fluid stimulation. Images were taken using an inverted microscope and the open area was calculated at the indicated time points using ImageJ software. (**C**) Graphs represents normalized (to t = 0 h) percent of wound healing at 24 h after initiation of a scratch in MCF7 and MDA-MB-468 cell lines stimulated with wound fluids. Data represent mean ± SD from 12 wound fluids for each group with statistical analysis corresponding to: *p < 0.05; **p < 0.01; ***p < 0.001, ****p < 0.0001.

A scratch assay was performed to determine the possible role of WF on the migration of breast cancer cells. Confluent MCF7 and MDA-MB-468 cells were incubated in the presence of the four WF groups (see Materials & Methods) for 48 hours. Next, the cells were wounded with a sterile pipette tip followed by wound healing (re-closure of the scratch) using light microscopy and incubated for another 24 hours. Images were taken at 0, 2, 4, 6, 8 and 24 hours after wounding. In the incubated MCF7 cell line cells, we found no significant differences between the incubated cells (four different mediums) in migration capacity to the wound area until the 8 h time point post-wounding compared to untreated cells (Fig. 3A,B). At 24 hours post-wounding, cells treated with WF presented a significantly higher migration capability. The percent of wound area in the WF-treated cells was significantly lower (0.278 ± 0.12) than in control cells (0.498 ± 0.08) and RT-WF treated cells (0.456 ± 0.14) (Fig. 3C). Notably, while there were no statistically significant changes between the WF and WF + RIBE groups, a difference in the size of the wound healing area was observed (WF = 0.278 ± 0.12, WF + RIBE = 0.415 ± 0.115) (Fig. 3C). The RIBE medium alone had no effect on the migration capability of MCF7 cells after 24 hours compared to control cells (WT = 0.498 ± 0.08, RIBE = 0.481 ± 0.12) (Fig. 3B,C). After WF stimulation (RT-WF, WF, WF + RIBE, and RIBE) a slight but non-significant enhancement in migration capability was observed at 4, 6, and 8 hours after wound initiation. However, after 24 hours, MDA-MB-468 cells migrated faster than control cells (0.424 ± 0.06) but only in the WF (0.194 ± 0.18) and WF + RIBE (0.325 ± 0.19) treated groups, although this difference in the WF + RIBE group was not statistically significant. Wound healing after WF stimulation (0.194 ± 0.18) was faster than in the RT-WF (0.491 ± 0.09) and WF + RIBE (0.325 ± 0.19) groups, with no significant difference between the latter two groups.

These findings clearly indicate that wound fluids harvested from breast cancer patients after BCS potentiate the migration capabilities of both MCF7 and MDA-MB-468 cell lines. However, this effect was abrogated if cells were incubated with WF from patients treated with BCS plus IORT (the RT-WF group). In the MDA-MB-468 cell line, the stimulatory effect of this fluid was also abrogated if WF-stimulated cells were additionally incubated with the RIBE medium.

## Discussion

Local relapse is one of the most important risk factors for metastasis and thus indirectly of worse survival among breast cancer patients following breast-conserving surgery^23^. Given that most local recurrences occur within the same quadrant as the primary cancer, a new therapeutic technique—IORT—has been introduced. The underlying rationale for IORT is that the immediate delivery of high dose irradiation could be more effective in inhibiting local recurrences than standard whole breast radiotherapy^24^. Follow-up reports from two IORT clinical trials (ELIOT and TARGIT) underscore the promise of this approach in the treatment of breast cancer^25^. However, high dose irradiation such as IORT has been shown not only to directly induce DNA damage in the irradiated cell, but also to induce changes in the tumor microenvironment^26^. Thus, in addition to its direct action, the effects of irradiation may also be observed in unirradiated cells located in close proximity, a phenomenon known as RIBE is observed in many cell types with several biological effects, including a significant modification of the tumor microenvironment^27,28^.

Both CSCs and inflammatory cells infiltrating the tumor can induce EMT^22^. Indeed, a strong correlation between chronic inflammatory conditions and tumorigenesis has been established^29^. Many researches proved, that WF treatment significantly induce breast cancer proliferation^30,31^. Moreover Krall *et al*. demonstrated that perioperative anti-inflammatory treatment reduces tumor growth and may reduce early metastasis in breast cancer patients^32^. WF are rich in cytokines, chemokines and matrix metalloproteinases and it has been proved that their composition depends on the biological features of the breast tumor that is removed^31,33^. In addition to promoting tumorigenesis, inflammatory cells such as tumor-associated macrophages (TAMs) and the factors they release (IL-1, TNF-α) are known to support all of the steps in the invasion and metastasis process, and thus such cells are known to induce EMT. Dong *et al*. showed that TNF-α induces Snai1 promoter activity and EMT transition in the MCF7 cell line, thus confirming the connection between inflammation and EMT^34^. Inflammation can also induce the CSC phenotype in breast cancer cells. Santisteban *et al*. have shown that CD8-positive T cells induce EMT in mouse mammary cancer cells; following activation, these cancer cells acquire a CSC phenotype (increased CD44^+^/CD24^low^ phenotype), drug resistance, and increased tumorigenicity^35^.

Given this context, the aim of the present study was to evaluate the mechanisms that influence the decreased tumorigenic potential of post-operative WF in patients treated with BCS and IORT. We hypothesized that wound fluids obtained postoperatively from patients after IORT would present a bystander effect (RIBE) that induces a radiobiological response in unirradiated cells and modifies their phenotype. We have previously investigated the connection between radiation-induced bystander effect and the anti-cancer properties of WF collected from BCS patients who underwent IORT. We observed that stimulation of breast cancer cells with RT-WF and with WF + RIBE resulted in higher level of double-strand breaks and increased expression of DNA damage repair-related genes in comparison with cells stimulated with WF. Thus we concluded that IORT induces a release of bystander factors, which mediate the genotoxic effect of radiation^20^. Moreover we showed, that WF collected 7 days after surgery from patients who underwent surgery alone, and those who received IORT affect the stem cell phenotype in breast cancer cell lines. We also showed a lower stimulation of CSC phenotype in IORT treated patients [15]. Thus, in order to elucidate whether this effect depends on RIBE and if fluids collected 48 hours after the surgery have a similar effect, wound fluids collected from patients after BCS alone (i.e., without IORT) were incubated with cells together with a RIBE medium and the phenotype of these cells was compared to WF from patients who underwent BCS plus IORT.

First, changes in the CSC phenotype were analyzed. As many researchers have reported, cancer cells displaying a stem-like phenotype play a crucial role in local recurrence, invasion, and metastasis as well as in radio- and chemo-resistance^36–38^. Al-Haij *et al*. identified a population of CD44^+^/CD24l^ow^ breast cancer cells believed to be more tumorigenic that other populations of breast cancer cells^39^. Our previous studies on WF collected from breast cancer patients one week after surgery have shown that postoperative fluids induce the CSC phenotype in the selected breast cancer cell lines. Moreover, in that previous study, we proved that IORT treatment reduces the tumorigenic potential of postoperative WF, as evidenced by the decreased CSC population^15^. Segatto *et al*. demonstrated that surgery-induced inflammation promotes stem-like phenotype and tumor initiating abilities of breast cancer cells^30^. Thus we wondered whether WF collected from breast cancer patients 48 hours after surgery would have a similar effect and if this effect could be abrogated by IORT treatment and co-incubation with the RIBE medium. The findings presented in this study indicate that the stimulatory effect of WF (WF group) on the CSC phenotype is partially abrogated by IORT treatment (RT-WF) and that this effect is dependent on the radiation-induced bystander effect. As the results of the TARGIT trial suggests, this effect may be dependent on an indirect role of cytokines present in postoperative fluids, which differ significantly between untreated and IORT-treated patients^40^. Belletti *et al*. analyzed the proteomic profile of postoperative fluids collected from breast cancer patients treated with and without IORT^8^, finding a group of cytokines with upregulated expression in the TARGIT-treated group; the expression of these cytokines (e.g., IL-4 and IL-5) is known to increase after radiation therapy^41–43^. Those same authors also demonstrated that many other factors— including IL-6, RANTES, HGF, STAT3—implicated in the control of cancer cell growth and motility and considered targets for anticancer therapies, were downregulated after TARGIT^8,44–46^.

Given that changes in the tumor microenvironment and CSC present in the tumor microenvironment may be responsible for inducing EMT in tumor cells^22^, we speculated that WF could induce EMT in luminal and basal/epithelial subtypes of breast cancer cells and that, based on the findings in our previous study, that this effect may be abrogated both by IORT treatment and co-incubation with RIBE medium. EMT is associated with enhanced aggressiveness and poor prognosis in carcinomas^47,48^. Changes from a more epithelial to more mesenchymal phenotype involve the systematic loss of epithelial markers (CDH1, EPCAM, keratins) and a gain in mesenchymal markers (CDH2, SNAI1, VIM). Functionally, cells which undergo EMT show increased migratory capabilities and an increase in the CSC phenotype^49^. Thus, we first analyzed expression of epithelial and mesenchymal markers in MCF7 and MDA-MB-468 cells incubated in the four different WF groups. Then, to functionally confirm the presence of the EMT program, we conducted a migration assay (i.e., the scratch assay). In both of the analyzed cell lines, we observed a induction of EMT process. Based on the expression of EMT markers, we confirmed the transition from epithelial to a more mesenchymal phenotype in WF-treated cells (in both the MCF7 and MDA-MB-468 cell lines). Interestingly, this effect was abrogated when cells were co-incubated with RIBE medium (WF + RIBE group), while no differences between the RT-WF and WF + RIBE groups were observed. These results clearly indicate the role of RIBE in WF collected from patients after IORT treatment. Given that the mesenchymal cells are characterized by enhanced motility, mainly through reduced adhesion forces, we performed an *in vitro* migration assay to functionally confirm EMT transition. We demonstrated that cells incubated with WF alone migrated much faster than RT-WF treated cells in both cell lines (MCF7 and MDA-MB-468) and that the migration was faster in the latter cell line. In addition, we showed that this stimulatory effect of WF on breast cancer cell migration was abrogated by the RIBE medium. Importantly, although those changes were observed in both cell lines, they were only statistically significant in the MDA-MB-468 cell line. These findings clearly indicate that the EMT marker expression (Figs 1 and 2) correlates with the migration capabilities of MCF-7 and MDA-MB-468 cells. Belletti *et al*.^8^ reported results regarding the stimulatory effect of postoperative fluids on the migration capabilities of breast cancer cells. Those authors found that the basal/mesenchymal MDA-MB-231 cell line in a 3D culture was invaded much faster after stimulation with WF from patients after BCS. The finding presented by Belletti and colleagues are consistent with our data, indicating that WF obtained from patients after IORT present a decreased migration capability. However, note that Belletti *et al*. used photon energy for IORT delivery whereas we used electron IORT. Nonetheless, given the similarity between our results, we can assume that the source of energy during IORT treatment does not affect the biological properties of the WF. As our experiments proved, the biological effect of WF after IORT treatment depends on RIBE, which induces a radiobiological response in unirradiated cells. EMT is a highly complex process with numerous different factors that can induce EMT in an *in vitro* culture. One factor that may be involved in this process is HGF (hepatocyte growth factor)^49^. Mostov *et al*. reported that HGF induces partial EMT in MDCK 3D cell culture and also pointed out that the HGF receptor (C-Met) has a higher expression in basal-like breast cancer cells^50^. As previously shown by Belletti and colleagues, HGF presents a decreased expression in WF collected from IORT versus non-IORT treated patients^8^. The findings reported by both Belletti *et al*. and Mostov *et al*. are consistent with our results, showing that not only do WF fluids stimulate breast cancer cells to undergo EMT at a much higher level than RT-WF treated cells (due to changes in the cytokine profile of WF) but also that this effect is more pronounced in basal-like breast cancer cells. Based on our results and other data reported in the literature, it appears that this effect is dependent on the cytokine profile of WF, which is altered by the radiation induced bystander effects initiated during IORT.

The present study demonstrates that wound fluids stimulate the CSC phenotype and EMT program in MCF7 and MDA-MB-468 breast cancer cell lines. This effect was partially abrogated when the cells were incubated in wound fluids collected from breast cancer patients after breast-conserving surgery followed by IORT treatment. Additionally, we confirmed the role of the radiation-induced bystander effect in altering the properties of the WF to induce the CSC phenotype and EMT program. The results presented here may be relevant not only to improve our understanding of the biological processes underlying changes in the properties of WF after IORT treatment, but may also be relevant for identifying effective treatments for breast cancer.

## Supplementary information

Dataset 1

## Data Availability

Data analyzed during this study are included in this published article. Any additional information are available from the corresponding author on reasonable request.
