# Supplementary material for: Wound fluids collected postoperatively from patients with breast cancer induce epithelial to mesenchymal transition but intraoperative radiotherapy impairs this effect by activating the radiation-induced bystander effect
Source: Sci Rep. 2019 May 27;9:7891. doi: 10.1038/s41598-019-44412-y (PMC6536501; doi:10.1038/s41598-019-44412-y)
Supplement: Supplementary file 1 — Dataset 1 [file 41598_2019_44412_MOESM1_ESM.pdf]

Supplementary information

Manuscript: ” Wound fluids collected postoperatively from patients with breast cancer induce epithelial to mesenchymal transition but intraoperative radiotherapy impairs this effect by activating the radiation-induced bystander effect”

Authors: Katarzyna Kulcenty, Igor Piotrowski, Karolina Zaleska, Mateusz Wichtowski, Joanna Wróblewska, Dawid Murawa, Wiktoria Suchorska

Supplementary Table 1. Patients characteristics

	**RT-WF patients group (n = 22)**	**WF patients group (n = 21)**
**Age at diagnosis Year ± SD**	58 ± 11,3	60 ± 10,6
**Molecular type (n)**		
Luminal A	8	7
Luminal B		
HER2-	8	7
HER2+	3	5
HER2	3	1

Supplementary Table 2. RT-qPCR values (mean of normalized mRNA level ± SD) presented on graph on Figure 2A

**MCF7**	**CTR**	**RT-WF**	**WF**	**WF+RIBE**	**RIBE**
CDH1	0.81 ± 0.15	0.45 ± 0.08	0.34 ± 0.02	0.45 ± 0.13	0.73 ± 0.18
EPCAM	0.76 ± 0.21	0.65 ±0.32	0.52 ±0.37	0.50 ±0.34	0.79 ±0.24
CDH2	1.05 ±0.19	0.89 ±0.40	1.45 ±0.49	0.92 ±0.47	1.25 ±0.36
SNAI1	1.05 ±0.25	0.55 ±0.35	1.01 ±0.54	0.6 ±0.32	0.75 ±0.34
VIM	1.12 ±0.13	0.72 ±0.39	1.24 ±0.43	0.59 ±0.40	1.24 ±0.19
**MDA-MB-468**	**CTR**	**RT-WF**	**WF**	**WF+RIBE**	**RIBE**
CDH1	0.95 ± 0.06	1.06 ± 0.45	0.47 ± 0.30	0.91 ± 0.43	0.84 ± 0.31
EPCAM	1.05 ± 0.19	1.28 ± 0.32	0.79 ± 0.28	1.16 ± 0.29	0.65 ± 0.24
CDH2	1.23 ± 0.49	1.16 ± 0.43	1.98 ± 0.90	0.99 ± 0.54	1.00 ± 0.17
SNAI1	0.68 ± 0.21	0.92 ± 0.29	1.42 ± 0.63	0.84 ± 0.21	0.48 ± 0.04
VIM	1.06 ± 0.13	1.14 ± 0.44	1.75 ± 0.76	1.00 ± 0.34	1.02 ± 0.31
